# Urban and Rural Safety Net Health Care System Clinics: No Disparity in HPV4 Vaccine Completion Rates

**DOI:** 10.1371/journal.pone.0096277

**Published:** 2014-05-09

**Authors:** Kelly Jo Sandri, Inge Verdenius, Mitchell J. Bartley, Britney M. Else, Christopher A. Paynter, Beth E. Rosemergey, George D. Harris, Gerard J. Malnar, Sean M. Harper, R. Stephen Griffith, Aaron J. Bonham, Diane M. Harper

**Affiliations:** 1 Department of Community and Family Medicine, University of Missouri, Kansas City School of Medicine, Kansas City, Missouri, United States of America; 2 Radboud University, Department of Obstetrics and Gynecology, Nijmegen, the Netherlands; 3 Department of Obstetrics and Gynecology, University of Missouri, Kansas City School of Medicine, Kansas City, Missouri, United States of America; 4 Hampshire College, Amherst, Massachusetts, United States of America; 5 Department of Biomedical and Health Informatics, University of Missouri, Kansas City School of Medicine, Kansas City, Missouri, United States of America; Banaras Hindu University, India

## Abstract

**Objective:**

Safety net health care centers in the US serve vulnerable and underinsured females. The primary aim of this work was to determine if HPV4 dosing compliance differs between females who receive doses at rural vs. urban core safety net health care locations.

**Methods:**

Females exclusively receiving health care in the Truman Medical Center (TMC) safety net system at the urban core and rural locations were identified by their HPV4 vaccine records. Dates and number of HPV4 doses as well as age, gravidity, parity and race/ethnicity were recorded from the electronic medical record (EMR). Appropriate HPV4 dosing intervals were referenced from the literature.

**Results:**

1259 females, 10–26 years of age, received HPV4 vaccination at either the rural (23%) or urban core location (77%). At the rural location, 23% received three doses on time, equal to the 24% at the urban core. Females seen in the urban core were more likely to receive on-time doublet dosing than on-time triplet dosing (82% vs. 67%, p<0.001). Mistimed doses occurred equally often among females receiving only two doses, as well as those receiving three doses.

**Conclusions:**

Compliance with on-time HPV4 triplet dose completion was low at rural and urban core safety net health clinics, but did not differ by location.

## Introduction

The Healthcare Research and Quality Act of 1999 (Public Law 106–129) has recognized the existence of health care disparities in the US. This means that participation in preventive services and cancer screening varies tremendously depending on patient residence: inner city/urban core vs. city/urban vs. rural vs. suburban [1]. In addition, health care disparities exist among the low income and non-white populations within the US [1]. While cervical cancer has been significantly reduced in the United States due to the organized Pap screening program, there are still significant differences in cervical cancer incidence by race: a higher incidence among Black and Hispanic women compared to white women [2], [3]. Likewise, there is a higher incidence of cervical cancer among poor and rural women [1]–[8] compared to women living in urban locations.

A decade ago significantly fewer women in rural areas underwent Pap screening than their urban counterparts [7], [9]. This disparity was successfully countered by establishing federally funded Community Health Centers (CHCs) that provided Pap testing along with complete health care: rural women receiving care at a CHC located in the same rural area had a significantly higher rate of Pap testing than did rural women who received health care in a local office [10]. While the rural CHC was successful in rural locations; urban CHCs still provided a higher rate of Pap testing to urban women [11]. Separate from the success of CHCs, though, Pap screening rates equalized between urban and rural dwellers, and between white and black females in many parts of the US by 2012 [12]. Disparities still exist, though, as rural women received HPV testing with their Pap testing significantly less often and were less likely to have public health coverage for screening compared to urban women [12]. In addition, while the data on HPV vaccination by geographic location is scant, early data appear to show lower uptake among rural females than urban with no data on compliance with dosing schedules [5], [12].

Women served by safety net health care systems are the most vulnerable for cervical cancer in the US [33]. These women often have no health care insurance or insufficient coverage for preventive services or cancer screening. Safety net health care systems serve both rural and urban core/inner city populations.

The primary aim of this study was to determine whether HPV4 dosing schedules differed between females who received care in rural and urban core safety net health care locations in Kansas City, Missouri during the most intense time frame for HPV4 vaccination advertising. The secondary aim was to determine what HPV vaccine dosing behavioral compliance pattern appears that could be used to design new intervention trials to reduce HPV prevalence in this population.

## Methods

This retrospective study was approved by the University of Missouri-Kansas City School of Medicine Adult Health Sciences Institutional Review Board (IRB) (#12–351) and no verbal or written consents were required.

All consents for review of the electronic medical record (EMR) were routinely obtained by the health care system for all patients seeking care in the system. All patients seeking care at Truman Medical Center (TMC) must sign a permission form for their EMR data to be used for any research or investigative purposes; hence the IRB approved this without any need for separate consent forms. Written consent was given from the next of kin, caretakers, or guardians on the behalf of the non-emancipated adolescents for their health care data to be stored in the EMR and used for research. The IRB waived active consent process for this study.

The main HPV4 vaccination study was conducted in the TMC safety net health care system to evaluate adherence to the three dose schedule [13]. TMC has two distinct physical locations serving mostly vulnerable low income populations. The time horizon for the study was July 1, 2006 through October 1, 2009, the dates during which HPV4 was the exclusively approved prophylactic HPV vaccine in the US. During this time frame, commercial advertising for HPV4 was intense. The EMR data pertinent to HPV4 vaccination included the location of vaccination, each date of vaccination and number of doses received. Personal descriptors included age at first vaccine dose, pregnancy history and race/ethnicity. Only females between 10–26 years of age were included in the study. The urban campus had one vaccination nurse who did not operate with a prepared script, but aggressively encouraged HPV vaccination of age eligible females; other than routine departure follow up instructions, there was no particular organized intervention to ensure timely completion of doses. The rural campus disseminated HPV vaccine through routine appointments.

HPV4 is approved in a timed three dose schedule [14], but has some limited immunologic evidence allowing consideration to a timed two dose schedule in those cases where compliance may be extremely difficult [15]–[17]. This work used the FDA approved three dose schedule as the primary endpoint of interest, but also explored the doublet dosing regimen for which there is efficacy for HPV2 [18]. HPV2 has efficacy against seven oncogenic HPV infections (HPV 16, 18, 31, 33, 45, 51 and 52) and cross protection providing a 30% efficacy against genital warts caused by HPV 6 and 11 [19]–[21].

Early dosing was defined as less than 4 weeks between dose 2 and dose 1; or less than 12 weeks between dose 3 and dose 2; or less than 24 weeks between dose 3 and dose 1. Late dosing was defined as more than 26 weeks between dose 2 and dose 1; or more than 52 weeks between dose 3 and dose 1 [13]–[17]. In other words, on time dosing between dose 1 and dose 2 was defined as more than 4 weeks and less than 26 weeks; between dose 2 and dose 3 was more than 12 weeks; and between dose 1 and dose 3 was more than 24 weeks and less than 52 weeks.

## Statistics

Student's t-test was used to compare means of continuous personal descriptors between the urban and rural locations; chi-square testing or Fisher's exact test was used to compare frequencies of race and number of HPV4 doses completed between females seeking care in the urban and rural locations [22]. Age groups were defined as adolescent: 10–18 years at first vaccine dose; vs. adult: 19–26 years at first vaccine dose. A singleton dose means that only one dose of HPV4 was received; a doublet dose means that only two doses of HPV4 were received; and a triplet dose means that three doses of HPV4 were received. We derived the Cochran-Armitage test for trend by using a transformation of the linear-by-linear association provided by SPSS [23], [24].

## Results

There were 9937 females seeking care exclusively at the rural location, and 17,849 seeking care exclusively at the inner city/urban core location during the study time frame. HPV4 initiation was significantly lower among 284/9937 (2.9%) of the rural eligible female population than 975/17849 (5.4%) of the urban females presenting for care (p<0.001, Table 1). Of those vaccinated in the TMC system, 975/1259 (23%) were seen at the rural location. Mean age, gravidity and parity of the females did not differ between the two locations. There were significantly more adolescents (10–18 years old) seen in the rural location than the urban core (28% vs. 22%, p = 0.033), but there were similar proportions of nulligravid (27% vs. 29%) and nulliparous (29% vs. 32%) females in both locations. Compared to the urban core location, the rural location served a greater proportion of white females (44% vs. 37%, p = 0.026), and a lesser proportion of black females (47% vs 54%, p = 0.021).

**Table 1:** Descriptors of females receiving care at the Inner City/Urban Core vs. Rural Safety Net Health Care Locations.

	Inner City/Urban Core		Rural		Total	
	N	Mean (SD)	N	Mean (SD)	N	Mean (SD)
Age, yrs	975	20.9 (3.4)	284	20.5 (3.7)	1259	20.8 (3.4)
Gravidity	933	1.4 (1.4)	268	1.5 (1.5)	1201	1.4 (1.4)
Parity	933	1.1 (1.1)	268	1.1 (1.1)	1201	1.1 (1.1)

Race	N	%	N	%	N	%
White*	358	36.7	125	44.0	483	38.4
Black§	529	54.3	132	46.5	661	52.5
Hispanic	55	5.6	15	5.3	70	5.6
Other	33	3.4	12	4.2	45	3.6

^*^There are significantly more white females in the rural location than urban, p = 0.026.

§ There are significantly more black females in the urban location than rural, p = 0.021.

There are no significant age or pregnancy history differences between the urban core population and the rural population.

The proportion of females receiving only one dose of HPV4 was similar between the rural and urban locations (42% vs. 40%) as seen in Table 2. The proportions of rural and urban recipients of only two and three doses of HPV4 were likewise not different. For both locations: there were significantly more females receiving a singleton dose of HPV4 than a doublet or triplet dose (p-for trend_rural_  = 0.006, p-for trend_urban_  = 0.031).

**Table 2:** Females receiving HPV4 by number of doses and Safety Net Health Center Location.

	Inner City/Urban Core N = 975		Rural N = 284		Total N = 1259	
	N	%	N	%	N	%
Number of HPV4 Doses						
n = 1	391	40.1	119	41.9	510	40.5
n = 2	238	24.4	77	27.1	315	25.0
n = 3	346	35.5	88	31.0	434	34.5
Appropriately Timed Triplet Series	231	23.7	64	22.5	295	23.4
Appropriately Timed Triplet Series among those receiving three doses		66.8		72.7		68.0
Appropriately Timed Doublet Series	195	20.0	57	20.1	252	20.0
Appropriately Timed Doublet Series among those receiving two doses		81.9		74.0		80.0

There are no significant differences between the urban core population and the rural population for any dosing scheme.

Among those at the urban core, significantly more completed two doses on time than three doses on time, 82% vs. 67%, p<0.001.

When appropriately timed dosing schedules are imposed, only 64/284 (23%) of females receiving care at the rural location received three on time doses: in a manner expected to result in maximal vaccine efficacies. Changing to those rural women who received three doses, 64/88 (73%) were able to complete the triplet dosing on-time. Similarly, of those rural females receiving only two doses, 57/77 (74%) were able to complete the doublet dosing on time. Among females at the urban location, 231/346 (67%) received triplet dosing on time, which was significantly lower than the proportion (195/238 (82%) who completed the doublet dosing on time.

The analysis of mistimed doses is presented in Table 3. Both urban core and rural populations received HPV4 dosing early equally often and late equally often for those completing both two and three doses.

**Table 3:** Mistimed HPV4 Doses.

	Inner City/Urban Core		Rural	
Triplet§	N = 346	%	N = 88	%
Early dosing	49	14.2	8	9.1
Late dosing	59	17.1	15	17.0
Both early and late dosing	7	2.0	1	1.1
Doublet*	N = 238		N = 77	
Early dosing	0	0.0	0	0.0
Late dosing	43	18.1	20	26.0

§ among those receiving three doses regardless of timing.

^*^among those receiving only two doses regardless of timing.

Early dosing, overall, occurs significantly more frequently among females receiving HPV4 at urban locations vs. rural locations (p = 0.03).

## Discussion

Our work shows that completion of HPV4 doses does not differ between vulnerable rural and inner city/urban core female populations. Females initiating HPV4 vaccination in an urban core safety net location complete the triplet series on-time as often as those in a rural safety net location, despite different site implementation activities: dedicated HPV4 nurse at urban location and routine clinic practice at rural location. This rate is similar to the reported adult HPV4 completion rates, but significantly lower than the general adolescent population completion rate [25]–[29].

While late doses were common, early doses carry larger implications for poor long term immunogenicity [14]–[17], [30]–[32]; in our study early dosing occurred only among the triplet completers. For this reason, and the superior completion rate of the doublet dosing among urban core HPV4 recipients, our study indicates that the urban core population may be better served by the doublet HPV2 series, which we have adopted. There appears to be no advantage to changing away from the FDA recommended HPV4 triplet dosing for the rural safety net subjects based on past vaccine behaviors reported in this study.

These two vulnerable sets of populations must be a high priority for reducing disparities in cervical cancer incidence. More than 60% of the cervical cancers occurring in the US develop in uninsured or underinsured women who live in medically underserved populations [33]. This bellwether of health disparities starts with a lack of health care access for preventive screenings. Rural women are less likely to undergo Pap screening, and more likely to present with advanced stage invasive cervical cancer than are women in more populated areas [3], [34], [35], whereas females in urban cores experience time lags for resolution of abnormal Pap screens [36]. Nonetheless, adolescents in rural populations, even with public insurance expansion such as the State Child Health Insurance Program (SCHIP), face lack of preventive services more often than their urban counterparts [37], [38]. Screening is the best method for early detection and prevention of cancer, but prophylactic HPV vaccination may offer protection to those at highest risk for abnormal Pap screenings [39], [40]. This study offers an early glimpse at the completed dosing schedules of HPV4 in rural and urban core populations.

Areas for future research include a modified HPV vaccination trial of HPV2 in two doses in females from the urban core, as they were behaviorally compliant most often with two on-time doses.

## Limitations

The limitations of the study methodology are several. This study only represents one Midwest US safety net health care system, and hence is not necessarily generalizable to other health care systems throughout the world. The two physical locations of the TMC safety net health care system are fixed. The inner city/urban core location continues to lose population and despite inner city rejuvenation, is forecast to continue serving a vulnerable and underinsured/uninsured clientele despite changes by the US Affordable Care Act. The rural location, on the other hand, is being subsumed by suburban growth at an accelerated pace. The years during which this study occurred had not yet seen the explosive suburban growth around this rural location, but undoubtedly there was some early influence of a more diversely insured clientele seeking care in the safety net health system.

The data for this study were extracted from the EMR and hence were limited to data that were routinely collected for clinic visits; billing data were not available for this analysis. Clinic visit data did not include level of education, occupation, place of residence or income levels; hence these factors could not be considered in this analysis.

While it is unlikely that the females seen in our safety net health care system received vaccination from other sources, it is possible that the initiation and completion rates are higher than we have in our EMR.

## Conclusion

Both rural and urban core safety net health care settings achieved the same low on-time completion rates of HPV4 vaccination.
